# Encapsulation of Piezoelectric Transducers for Sensory Augmentation and Substitution with Wearable Haptic Devices

**DOI:** 10.3390/mi8090270

**Published:** 2017-09-02

**Authors:** Francesca Sorgini, Alberto Mazzoni, Luca Massari, Renato Caliò, Carmen Galassi, Sunil L. Kukreja, Edoardo Sinibaldi, Maria Chiara Carrozza, Calogero M. Oddo

**Affiliations:** 1 The BioRobotics Institute, Scuola Superiore Sant’Anna, Viale Rinaldo Piaggio 34, 56025 Pontedera, Italy; alberto.mazzoni@santannapisa.it (A.M.); luca.massari@santannapisa.it (L.M.); renato.calio@santannapisa.it (R.C.); chiara.carrozza@santannapisa.it (M.C.C.); 2 Istituto di Scienza e Tecnologia dei Materiali Ceramici (CNR-ISTEC), Via Granarolo, 64, I-48018 Faenza, Italy; carmen.galassi@istec.cnr.it; 3 Singapore Institute for Neurotechnology (SINAPSE), National University of Singapore (NUS), 28 Medical Dr. #05-COR, Singapore 117456, Singapore; sunilkukreja.sinapse@gmail.com; 4 Center for Micro-BioRobotics, Istituto Italiano di Tecnologia, Viale Rinaldo Piaggio 34, 56025 Pontedera, Italy; edoardo.sinibaldi@iit.it

**Keywords:** polymeric matrix, piezoelectric, touch psychophysics, vibrotactile stimulation, bi-finger perception, sensory substitution, sensory augmentation, tactile display, tactile telepresence

## Abstract

The integration of polymeric actuators in haptic displays is widespread nowadays, especially in virtual reality and rehabilitation applications. However, we are still far from optimizing the transducer ability in conveying sensory information. Here, we present a vibrotactile actuator characterized by a piezoelectric disk embedded in a polydimethylsiloxane (PDMS) shell. An original encapsulation technique was performed to provide the stiff active element with a compliant cover as an interface towards the soft human skin. The interface stiffness, together with the new geometry, generated an effective transmission of vibrotactile stimulation and made the encapsulated transducer a performant component for the development of wearable tactile displays. The mechanical behavior of the developed transducer was numerically modeled as a function of the driving voltage and frequency, and the exerted normal forces were experimentally measured with a load cell. The actuator was then tested for the integration in a haptic glove in single-finger and bi-finger condition, in a 2-AFC tactile stimulus recognition test. Psychophysical results across all the tested sensory conditions confirmed that the developed integrated haptic system was effective in delivering vibrotactile information when the frequency applied to the skin is within the 200–700 Hz range and the stimulus variation is larger than 100 Hz.

## 1. Introduction

In recent years, the development of haptic devices for different application purposes has increased. The growing spread of tactile displays is due to the high potential of the tactile sense as a communication channel for the remote transmission of information in a variety of situations. Due to the high number of tactile receptors located on our skin, particularly on the hands [1,2], the sense of touch represents a means to deliver information, which can also come from other sensory modalities such as vision and audition in sensory-disabled subjects [3,4,5,6,7]. In this case, the information coming from one sensory channel is conveyed to the tactile sense in an understandable way.

Technologies based on different actuation principles were integrated in haptic feedback systems. Polymeric actuators, like electro-active polymers, have been used for the development of tactile displays for rehabilitation or virtual reality applications [8]. The ability of dielectric polymers to undergo large displacements [9,10] and their muscle-like behavior made them suitable for the integration in haptic devices. Applications include tactile displays for the communication of textual and graphical information for blind persons, where electroactive silicone polymers were used for the development of planar arrays of pins [10,11]. These actuators were also integrated into tactile displays for the blind. A relevant example is a finger-tactile display initially designed to stimulate fingertips, made of a soft polymer which includes a matrix of dielectric elastomer dots that expand or compress transmitting information to the fingers [12]. Arrays of piezoelectrically-activated pins were also designed for the development of rewritable Braille cells, where a polydimethylsiloxane (PDMS) membrane encapsulated an incompressible fluid [13,14]. Electro-active polymers for virtual reality applications were used to develop haptic interfaces, like mice, joysticks, trackballs, gamepads, steering wheels, styluses, tablets, and pressure-sensitive spheres [15].

The polymers’ properties of softness and compliance, together with the easiness of their shaping with dedicated and customizable polymerization processes, make them a useful matrix for the encapsulation of transducers based on other actuation principles. A polymeric encapsulation can also facilitate the integration of the transducers in more complex haptic systems, i.e., wearable tactile displays for sensory substitution and/or augmentation in contexts like manufacturing, virtual reality, and rehabilitation. Anyway, the use of polymers as a soft interface between a transducer and the external environment is more common for the fabrication of sensors, especially tactile sensors based on MEMS, electrodes, or polyvinylidenefluoride (PVDF) sensing technology [16,17,18,19,20,21].

Largely employed in the development of interfaces for haptic feedback are also vibrotactile actuators. Vibrotactile stimulation can be used for sensory substitution in sensory-disabled, or for sensory augmentation in non-disabled, individuals. Haptic stimulation for force feedback can, in fact, improve object manipulation tasks in virtual environments, and can be largely employed for rehabilitation, navigation, rescuing, and robot remote control purposes [22,23,24,25,26]. Haptic displays were also proven useful in robotic surgery and in industrial environments, enhancing human-robot co-working. They can, in fact, allow the development of alerting devices in all those contexts where the interaction with automated machinery can be dangerous.

Different vibrotactile wearables can be found in the literature, designed for the various purposes already mentioned before, and actuated with technologies like pneumatic and piezoelectric actuators, vibrating motors, or solenoids [27]. Vibrotactile gloves were designed to allow deaf-blind individuals to communicate with the not-disabled community [28], or to improve manipulation tasks in virtual reality applications [29]. Belts and vests were developed to give vibrotactile feedback in virtual reality (VR) applications [30,31], or to assist the navigation of visually impaired individuals [32,33], as were vibrotactile wristbands [34]. Prototypes of head-mounted devices were developed to haptically guide the user in unknown environments via tactors [35,36].

In the following, we focus on the development and evaluation of a piezoelectric transducer encapsulated in a polymeric matrix, and on its integration in wearable haptic displays to deliver information via the tactile sense, starting from the hypothesis that the introduction of a compliant interface between the stiff piezoelectric element and the soft human skin can have an influence at a perceptual level. In previous studies a polymeric layer has usually been introduced to cover an array of active elements [12,37], or a tactile sensor [16,17,18,19,20,21]. Our customized fabrication procedure allows to obtain an encapsulated element that enables the development of a single encapsulated actuator, scalable in size, with a very good match between modelled transduction and the actual prototype, and which can be integrated in a straightforward manner in wearable haptic devices for the stimulation of different body areas on single or multiple contact points. We found that the geometry and the material selected for the encapsulation of the transducer resulted in a system capable of reliably delivering vibrotactile information, where this was confirmed both on the side of electromechanotransduction behavior and on the human somatosensory perception.

The following sections of this paper present the design and fabrication procedure for the developed vibrotactile haptic transducer with polymeric encapsulation, the finite element method (FEM) modelling of its transduction properties, and the procedures for its experimental evaluation (Section 2); then, the results are provided and discussed with respect to the modelling and its assessment via bench tests, and the transducer is used within a psychophysical protocol involving healthy subjects (Section 3); finally, the conclusions draw the perspectives for future applications of the system (Section 4).

## 2. Materials and Methods

### 2.1. Piezoelectric Encapsulated Transducer

The implemented solution consists in the integration of a piezoelectric disk (7BB-12-9, MuRata, Kyoto, Japan), 12 mm in diameter and 220 µm in thickness, in a polymeric matrix (polydimethylsiloxane, PDMS, Dow Corning (Midland, MI, USA) 184-Silicone Elastomer).

The PDMS encapsulation serves both the mechanical and electrical roles. It allows electric contacts to be encapsulated, providing electrical insulation of the element. In addition, it allows obtaining a system that can be easily inserted in wearable haptic devices, such as gloves and wristbands for the upper-limb stimulation, or ankle bands and insoles for the lower-limb stimulation. The compliance of the polymeric encapsulation constitutes also an adaptation interface between the stiffness of the transducer (Piezo devices made of lead zirconate titanate (PZT) on a brass mass) and the softness of the human skin.

The development of the encapsulated transducer is articulated in different steps.

**Figure 1:**
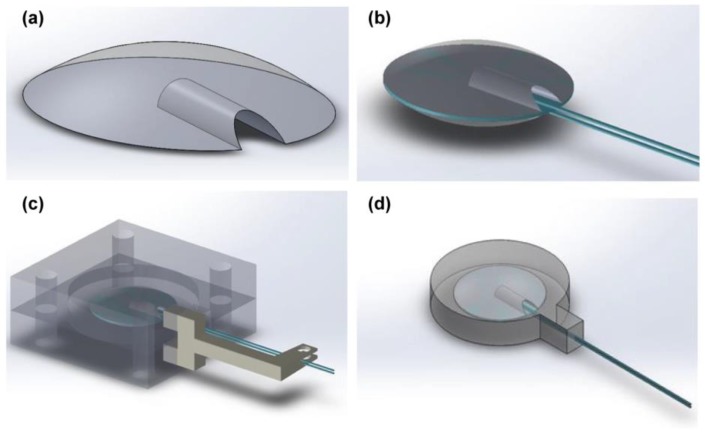
Encapsulated transducer for haptic applications. (**a**) Upper view of one of the spherical cups; (**b**) Encapsulated transducer with the two spherical cups on the opposite sides and the embedded electrical contacts; (**c**) 3D-printed customized mold for the development of the geometry of the transducer with PDMS polymer. The piezoelectric element with the spherical cups and the electrical connections are located at the center of the molding stucture; and (**d**) an upper view of the transducer, with evidence of the internal structure where two spherical cups enclose the piezoelectric disk and the electrical wires.

The first step is the development of two polymeric (PDMS) elements in the shape of spherical cups with the same diameter of the piezoelectric disk 2 mm in height (Figure 1a). These elements are obtained by a casting process in customized 3D-printed moulds. Elements are also provided with a housing on the flat side, designed to contain the electrical wires connected to the piezoelectric disk. In order to fix the wires on the transducer, the spherical cups are placed on the opposite sides of the piezoelectric disk after the application of a conductive epoxy (CircuitWorks conductive epoxy—Chemtronics (Kennesaw, GA, USA)) (Figure 1b), and the whole resulting system is closed in a further 3D printed customized mould for the final polymeric encapsulation, which will be made of the same material (Figure 1c).

The role of the polymeric spherical cups protruding from each side of the external surface is to keep the piezoelectric element centred in the encapsulating polymer (Figure 1d). At the same time, they create two bumps on the external sides of the element in order to focus the transducer deformation, the vibrotactile stimulus, in a specific contact area on the skin.

The choice of a spherical cup as a contact region on the skin is due to heuristic design criteria. On one side, a spherical protrusion simplifies the implementation of some steps of the encapsulation process. Furthermore, protruding edges [19] or bumps [12,13,14] showed proper functionality in the design of matrices of tactile actuators, and bumps are the most used in Braille displays. We opted for a spherical-like shape to avoid sharp edges on the actuator surface and to increase the comfort of the wearer. The dimensions were chosen in order to have a perceptible protrusion which could help position the actuator on a specific contact point on the skin, but also to avoid a very large difference in height from the actuator surface. The resulting contact area was about 18.2 mm^2^ which, in case of multiple transducers, results in a spatial distribution just higher than the minimal range guaranteeing reliable two-point discrimination on fingertips (0.5 mm [38]–1.6 mm [39]).

### 2.2. FEM Model of Transducer’s Electro-Mechanical Behaviour

A finite element method (FEM) simulation of the piezoelectric transducer was performed using COMSOL Multiphysics (COMSOL Inc., Palo Alto, CA, USA). We considered the geometry introduced in Section 2.1. The mechanical properties of PDMS necessary to run the simulations were the Young’s modulus, Poisson’s ratio, and density. The acoustic properties were not explicitly needed as inputs for the simulation. They were consistently derived by the numerical solver so that no additional characterization was required. The density was verified from the mass/volume ratio of auxiliary samples. The derived value was in agreement with the one available in the adopted library (970 kg/m^3^). Regarding the PDMS Poisson’s ratio, we adopted 0.5 from the literature [40,41] (its dispersion is low, and the same value was provided as the reference PDMS Poisson’s ratio in the material library of the chosen solver). Differently, the PDMS Young’s modulus sensibly depends on the actual material composition and therefore we assessed the reference value provided in the material library through a complementary calibration experiment described in Section 2.3. Finally, as for the PZT material properties, we adopted the elastic and piezoelectric coefficients and electric permittivity provided by the material library of the chosen solver (an extensive characterization of this commercial material was beyond the present scope).

The purpose of the numerical simulations was to estimate the normal force exerted by the piezoelectric transducer at different frequencies and driving voltages. In particular, we chose 50 V, 100 V, and 150 V as the driving peak-to-peak voltages for the sake of definiteness, and we selected a frequency range between 200 Hz and 700 Hz. The choice of such a frequency interval allows the activation of the transducer within the Pacinian frequency range, centred around 300 Hz [3,39,42,43], for which the maximum sensitivity for vibrotactile stimulation is expected. Furthermore, previous studies [44] showed that the subjective amplitude perception of the vibrotactile stimulus is not influenced by the frequency in this specific interval (200–700 Hz).

**Figure 2:**
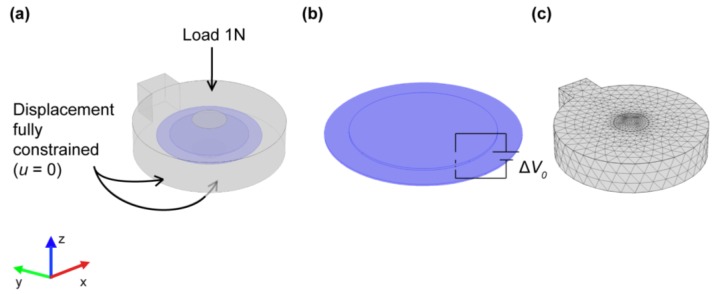
Finite element method (FEM) model of the encapsulated transducer. (**a**) Schematic of the actuator showing the boundary conditions on the PDMS structure; (**b**) detail of the piezoelectric disk showing the driving voltage imposed on the element; and (**c**) a view of the meshed geometry of the whole encapsulated transducer.

Based on the experimental conditions described below (Section 2.4.1), we imposed a null displacement (*u* = 0) on the bottom and lateral surface of the PDMS encapsulation material, whereas we imposed a compression load of 1 N on the PDMS upper surface (see Figure 2a). We imposed the driving voltage on the piezoelectric disk (ΔV0), and we gathered via a load cell (Nano 43, ATI Industrial Automation, Apex, NC, USA), the resulting force normal to the PDMS bottom surface. In addition, we preliminarily set up the numerical discretization so as to obtain grid-independent results, which led to a mesh composed of 675,062 tetrahedral elements (160,731 volume elements, 23,068 surface elements, and 843 edge elements). We also considered an axisymmetric model, derived from the three-dimensional one through minor geometrical simplifications, to assess the adopted numerical technology. For all the runs, we considered the fully-coupled (i.e., electro-mechanical) problem by exploiting the corresponding modules natively provided with the FEM simulation environment.

### 2.3. Preliminary Mechanical Characterization of the PDMS

A mechanical characterization of the PDMS was performed in order to assess the Young’s modulus. A cylindrical probe (φ 6 mm) moving along the Z-axis through a motorized translational stage was used to indent a PDMS sample (30 mm × 30 mm × 3 mm). The probe was mechanically linked with a load cell (Nano 43, ATI Industrial Automation) in order to apply a predefined value of force and, thus, establish a relationship between the applied force and the corresponding polymer displacement (indentation; set to be null when contact was first established). The resulting experimental trend was then compared to the one obtained from a numerical simulation of the considered indentation test, exploiting, in particular, the Young’s modulus provided by default by the adopted numerical solver.

### 2.4. Experimental Electromechanical Characterization of the Encapsulated Transducer

#### 2.4.1. Experimental Setup

**Figure 3:**
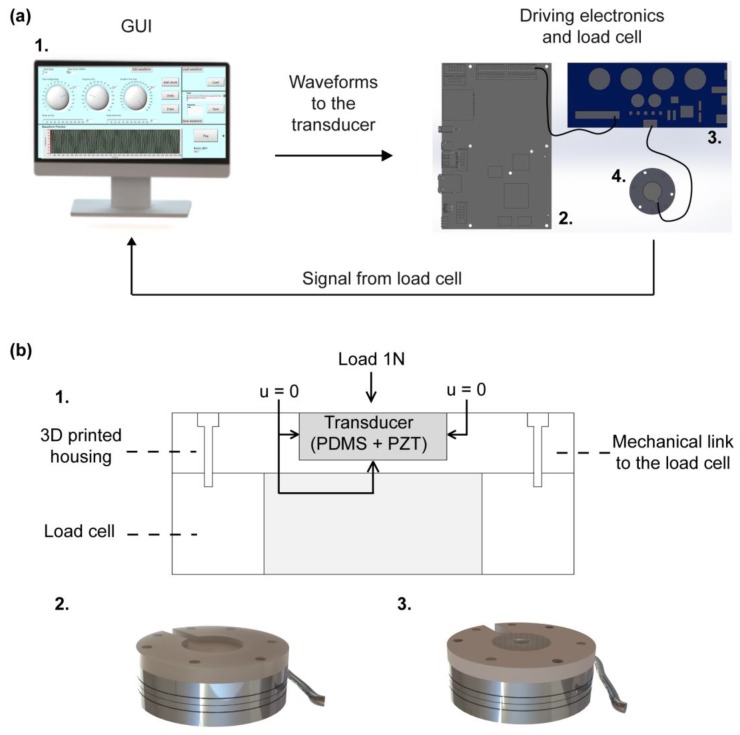
Experimental setup for the evaluation of the normal force exerted by the encapsulated transducer. (**a**) Schematic drawing of the experimental setup: 1.) PC running a GUI to send selected waveforms to the piezoelectric transducer via a driving electronics; 2.) electronic board for the communication between the GUI and the piezo haptic driver; 3.) piezo haptic driver for the activation of the piezoelectric transducer; and 4.) load cell for force measurement: the measured forces are saved for post-processing. (**b**) Detail of the mounting for the electromechanical characterization of the transducer, where the encapsulated transducer is fixed on the load cell: 1.) Section of the measurement system, in which the encapsulated transducer in fixed in a 3D printed housing linked to the load cell, with detail of the experimental boundary conditions; 2.) 3D view of the experimental setup for the electromechanical characterization of the transducer, with a 3D printed housing linked to the upper part of the load cell; and 3.) 3D view of the whole measurement system, in which the encapsulated transducer is inserted.

In order to perform the mechanical characterization, the transducers were actuated by means of a piezo haptic driver (DRV2667 Evaluation module, Texas Instruments, Dallas, TX, USA) using a graphical user interface (GUI) (LabVIEW, National Instruments, Austin, TX, USA) that activated the driver through an electronic board (sbRIO 9636, National Instruments). Before human evaluation of the system, we assessed the ability of the haptic interface to deliver perceptible and discriminable stimuli using a load cell (Nano 43, ATI Industrial Automation), in order to provide input stimuli and record the resultant vibrations (Figure 3b) [45]. Such measurements were then compared with the FEM simulations described in Section 2.2.

#### 2.4.2. Experimental Procedure

The experimental mechanical characterization of the encapsulated transducer was performed in order to evaluate the element behaviour with the variation of the driving voltage (measured in peak-to-peak-Vpp) and the frequency. In order to do so, we measured the amplitude of the normal force (Fz) exerted by the piezoelectric element on a load cell. To stabilize the transducer during the experimental tests, a 3D-printed housing was fixed on the load cell (Figure 3b). Furthermore, a load of 1 N was placed on the upper surface of the encapsulated transducer in order to keep it stable during the measurements and to emulate a typical pre-load that can be exerted on the device during its use. Such an offset load was then subtracted from the dynamic measurements of the load cell.

The piezoelectric element was driven with stimuli lasting 1 s. The stimulation signals were characterized by three values of amplitude (50, 100, and 150 Vpp), kept constant across each stimulation, and 21 values of frequency varying between 200 Hz and 700 Hz, with 25 Hz steps. These settings were consistent with those adopted for the model, mentioned in Section 2.2. The values of the normal force (Fz) exerted on the load cell during the excitation were acquired across 10 repetitions for each vibration frequency and each peak-to-peak voltage.

#### 2.4.3. Data Analysis for the Electromechanical Characterization of the Encapsulated Transducer

The waveforms obtained from the measurements of the load cell were then analysed with the calculation of the signal power (standard deviation of the amplitude of Fz). The data analysis was performed across 750 samples for each frequency value, where the sampling window was selected in the central part of the signal to focus on the steady state of the dynamic activation of the piezoelectric transducer.

Spectral analysis was performed on Fz using the MATLAB (R2016b, MathWorks, Natick, MA, USA) wavelet coherence package [46], for each peak-to-peak voltage and each frequency value in the range between 200 Hz and 700 Hz, with 25 Hz steps and 50 Hz steps (see Section 3.3).

### 2.5. System Integration for Psychophysical Evaluation

**Figure 4:**
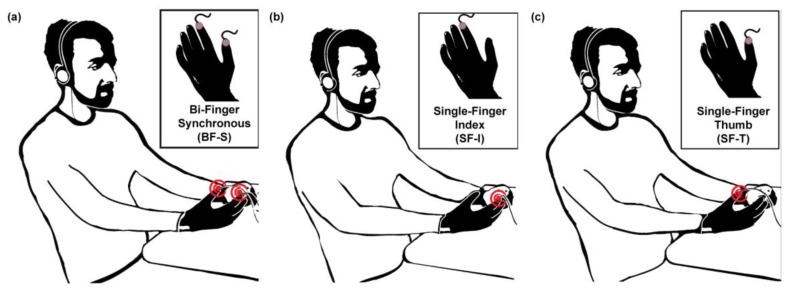
Experimental setup with the three experimental configurations. (**a**) Bi-finger synchronous (BF-S) configuration: two piezoelectric transducers, embedded in a spandex glove, synchronously stimulate the tips of the index and thumb finger; (**b**) single-finger index (SF-I) configuration: single-finger stimulation on the index fingertip with one piezoelectric transducer embedded in a spandex glove; and (**c**) the single-finger thumb (SF-T) configuration: stimulation on the thumb fingertip with one piezoelectric transducer embedded in a spandex glove.

The described encapsulated transducer was used for the development of a wearable vibrotactile device for the stimulation of the hand, i.e., a haptic glove (Figure 4). For the psychophysical evaluation of the tactile display, we designed three experimental configurations: two single-finger, with the stimulation of the index (SF-I) or thumb (SF-T) fingertip; and one bi-finger (BF-S), with the simultaneous stimulation of the index and thumb fingertips. In the SF-I and SF-T configurations, one transducer was integrated, respectively, on the tip of the index finger (Figure 4c) or thumb (Figure 4d) of a spandex glove; for the BF-S configuration two transducers were integrated on the tips of the index finger and thumb of the same spandex glove (Figure 4b). In all configurations, the glove allowed a secure positioning of the vibrating element on the participant’s fingers. The transducers provided a contact area with the finger pad of approximately 250 mm^2^. The electrical actuation was delivered to the transducers via the electronics already described in Section 2.4.1, i.e., a piezo haptic driver controlled using a GUI and interfaced with the driver through an electronic board [45].

### 2.6. Psychophysical Evaluation

We evaluated the ability of the integrated wearable haptic system to deliver accurate tactile feedback using a two-alternative forced choice (2-AFC) psychophysical protocol. According to previous studies, we chose to use frequency modulation as a mean to deliver haptic information. Even if the exact number of discriminated levels is not clear yet, it can increase when stimuli differing in frequency are relatively compared [43]. Studies regarding the amplitude modulation showed instead that, for constant frequencies, when the vibration amplitude increases, the perceived frequency also increases [47]. 

According to these studies, and to the experimental data from our measurements, we decided to select a fixed driving voltage of 150 Vpp for the psychophysical experiments. This value corresponds to the higher value of normal force exerted on the actuators’ sides and, according to preliminary psychophysical tests, was the one which showed the best performance across 10 participants [45]. The frequency modulation was then performed in the range between 200 Hz and 700 Hz, that guarantees a proper functioning of the transducer according to the FEM analyses (see Section 2.2 for methods and Section 3.2 for results). 

#### 2.6.1. Participants

Thirty-three healthy participants (15 females and 18 males), aged between 25 and 37, participated in psychophysical experiments. Haptic stimulation was performed on the dominant hand which, for 31 participants, was the right hand. No participant had previously performed any activity presumably compromising finger tactile sensitivity. All participants provided written informed consent for inclusion before they took part in the study. The study was conducted in accordance with the Declaration of Helsinki, and the protocol was approved by the Ethics Committee for non-clinical experimentation of Scuola Superiore Sant’Anna of Pisa.

#### 2.6.2. Experimental Procedure

A tactile discrimination task with the 2-AFC procedure [48] was designed, and was performed by each participant. Periodic vibrotactile stimulation was delivered using the haptic glove described in the previous section. The experimental session consisted of the presentation of 150 pairs of stimuli divided into 15 sequences, as described in the following.

**Figure 5:**
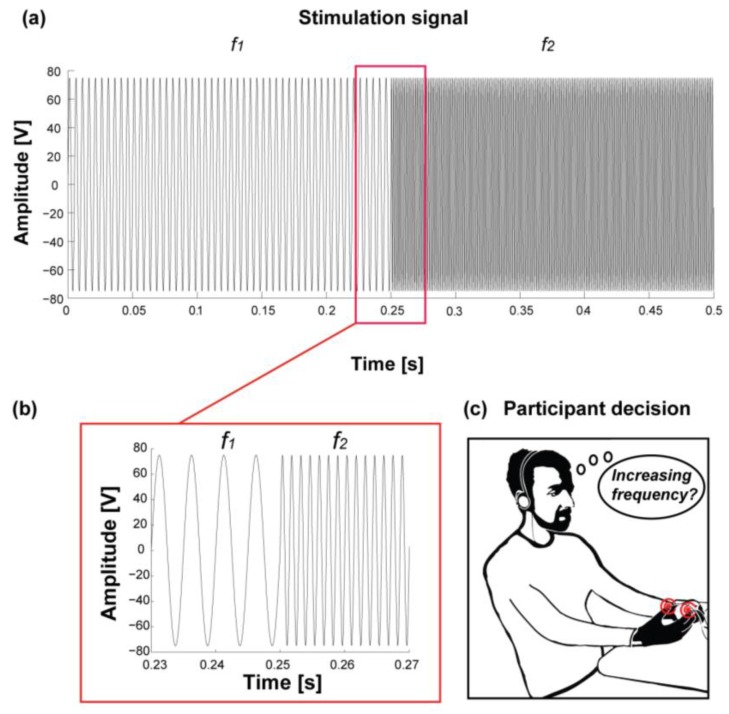
Stimulation and task. (**a**) An example of a pair of stimuli: 250 ms of sinusoidal oscillations with a frequency of 200 Hz followed by 250 ms sinusoidal oscillation with a frequency of 700 Hz. The peak-to-peak amplitude activating the transducer was fixed at 150 V; (**b**) a 0.04 s slice of the vibrotactile stimulation shown in panel (**a**), depicting the frequency transition at 0.25 s; and (**c**) the participant decision phase: after perceiving the vibrotactile stimuli pair, the participant was asked to determine whether the first or the second had the higher frequency content.

Each participant was presented with paired vibrotactile stimuli (Figure 5a,b) and was asked to identify which stimulus of the pair had the higher frequency content (Figure 5c). A stimulation sequence included a single presentation of each of the 10 pairs of stimuli described in Table 1 in random order. An 8 s interval was introduced to separate each subsequent stimuli pair, leading to a sequence duration of about 2 min. A rest period of about 1 min spaced the 15 sequences, for a total average duration of 45 min. Two randomized sequences were used for training purposes. These sequences were not included in the statistical analyses and were sufficient for the participants to familiarize with the stimuli.

As briefly explained in the introduction, we delivered vibrotactile stimuli following three experimental configurations. In this way, we were allowed to test the human hand tactile sensitivity to frequency variations under different perceptual conditions. Each configuration was tested with 11 participants. Each participant was comfortably seated on a chair for the duration of the experiment for all tested configurations, and he/she was acoustically isolated from the environment with white noise provided by headphones.

#### 2.6.3. Data Analysis for Psychophysical Experiments

Data analysis was performed using the Statistics Toolbox in MATLAB. To compare the performance of different configurations, the Kruskal-Wallis test was used. For each configuration and frequency variation, the vibrotactile perception of a population of participants was evaluated by the median and the 95% confidence interval of the rates of identification of stimuli having an increasing frequency (Δ*f* > 0), calculated with binofit test. A logistic fit of the resulting psychometric curves was computed for each configuration across presented frequency variations. To analyse the significance of participants responses for each frequency variation Δ*f* and experimental configuration, the binofit test was used.

## 3. Results and Discussion

### 3.1. Encapsulated Piezoelectric Transducer

**Figure 6:**
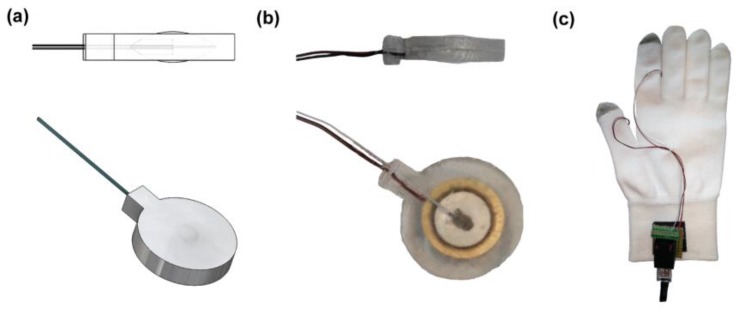
Embedded piezoelectric transducer. (**a**, **upper part**) Drawing of the lateral view of the piezoelectric transducer embedded in the polymeric matrix, with evidence of the two protrusions on the external opposite faces of the geometry; (**a**, **lower part**) drawing of the upper view of the piezoelectric transducer embedded in the polymeric matrix; (**b**, **upper part**) lateral picture of the developed prototype showing the side of the actuator; (**b**, **lower part**) upper picture of the developed prototype showing the whole surface of the actuator; and (**c**) a picture of the integrated system used for the psychophysical evaluation: a textile glove equipped with two encapsulated transducers on the thumb and index fingertips.

We developed an encapsulated transducer, 18 mm in diameter and 4 mm in thickness (Figure 6) (see methods for details). Its shape is characterized by two spherical cups which protrude out 250 µm from the upper and lower levels of the polymeric matrix (upper part of Figure 6a,b). These elements allow skin stimulation at a specific contact point. The presence of the spherical cups allows to centre the piezoelectric element in the polymeric shell, as shown in the lower part of Figure 6b.

### 3.2. Results of the Preliminary Experimental Mechanical Characterization of the PDMS

**Figure 7:**
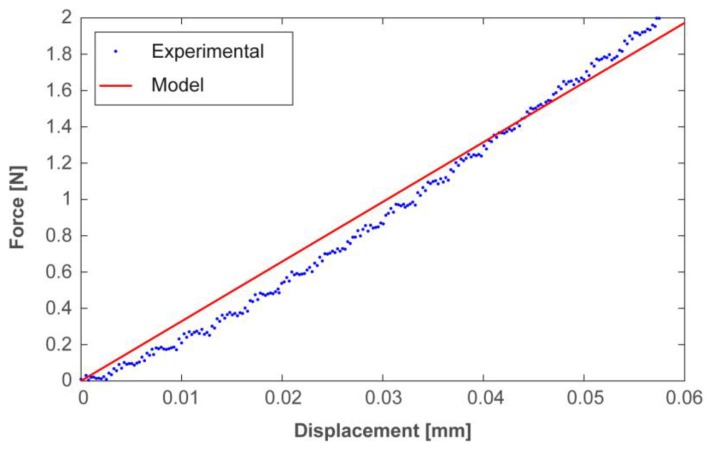
Characterization of the stiffness of the fabricated PDMS test samples and corresponding model calibration. The agreement between experimental (blue dots) and simulated data (red line) confirmed the suitability of the chosen model parameters.

Results from the experimental characterization of PDMS (see Section 2.3) validated the stiffness of PDMS test blocks, maintaining the mechanical parameters of the material as available in the software library. In particular, the trend of the obtained force vs. displacement was compared with the one obtained from the model of the considered indentation test. The simulation outcomes appear to match the experimental data, to consider the library parameters of the material accurate enough to describe the PDMS used in our study (Figure 7). As a consequence, we could safely exploit the parameters available in the COMSOL material library to run the FEM dynamic simulations presented in the next section.

### 3.3. Transducer FEM Model and Experimental Characterization

**Figure 8:**
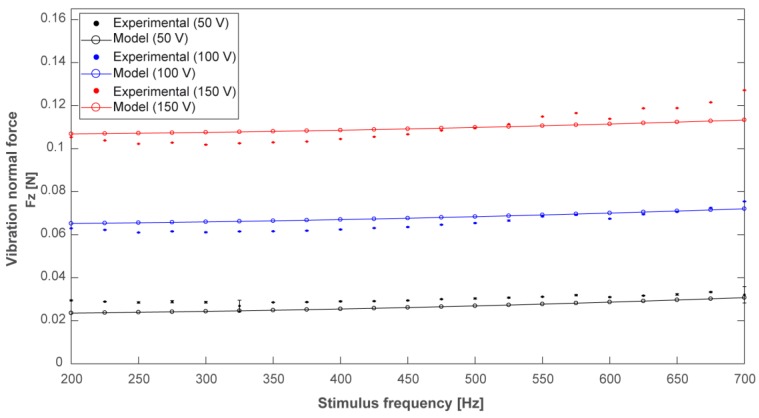
Vibrational component of the normal force exerted during transducers actuation. Predicted trend (solid lines with circles) and experimental measures (dots) of the normal force for the considered driving voltages. For each experimental point, the standard deviation is represented by vertical bars.

The results of the FEM model are shown in Figure 8 (solid lines with circles), together with the corresponding experimental measures (dots). The model was able to accurately predict the observed experimental response. For each driving voltage and each frequency, the mean value and the standard deviation of the normal force (over the 10 repetitions) are represented in Figure 8. In most cases, the vertical bars representative of the standard deviation are hardly visible thanks to the high repeatability of the experimental conditions.

From Figure 8 we can conclude that, as expected, the amplitude of the vibrational component of the normal force increases with the increase of the transducer driving voltage. Figure 8 also shows how the vibration amplitude is weakly varying over the analysed frequency range for each driving voltage (for 150 Vpp driving voltage, the variation of the normal force amplitude is about 0.025 N across the selected frequency range). These results demonstrated that the variation of the normal force is mainly related to the driving voltage amplitude. Increasing the voltage value (from 50 V to 150 V) three times leads to a fractional variation of the recorded force of about 300% ± 25% over the whole range of frequencies. Increasing the frequency value three times (from 200 Hz to 600 Hz) leads, instead, to a force variation always lower than 10% for all of the driving voltage conditions (50 V, 100 V, and 150 V). The weak relationship from the value of frequency within the selected range allows a straightforward application of the transducer in haptic displays for the stimulation of the human hand.

**Figure 9:**
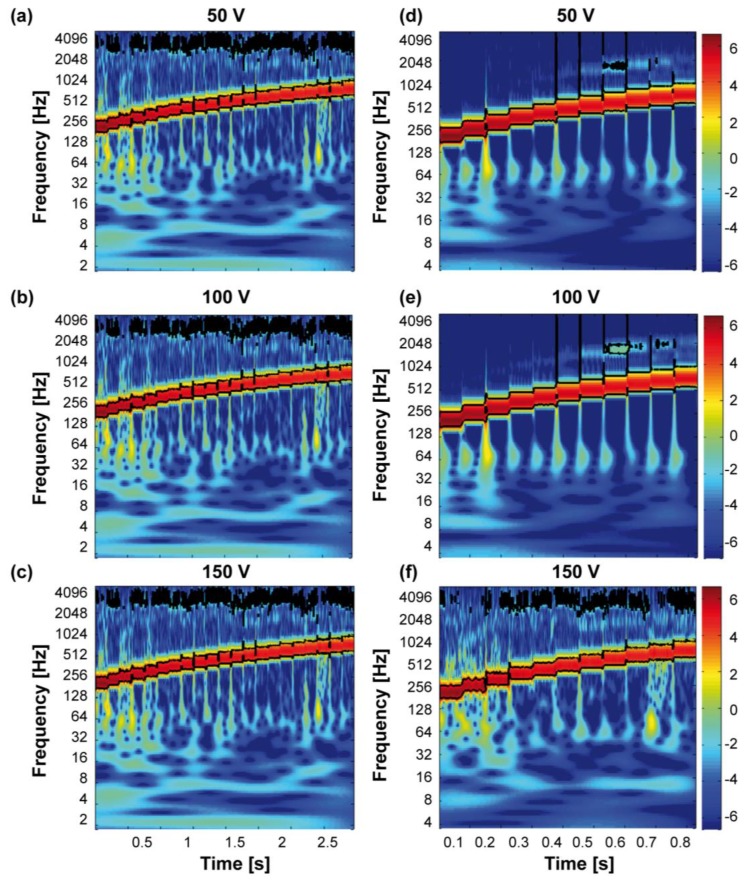
Frequency power spectrum of the normal force. Spectral analysis of the normal force recorded by the load cell while activating the piezoelectric actuator/polymer system. (**a**) Results for 50 Vpp and 200–700 Hz, with 25 Hz steps (electromechanical characterization); (**b**) results for 100 Vpp and 200–700 Hz, with 25 Hz steps (electromechanical characterization); (**c**) results for 150 Vpp and 200–700 Hz, with 25 Hz steps (electromechanical characterization); (**d**) results for 50 Vpp and 200–700 Hz, with 50 Hz steps (pilot psychophysical experiments); (**e**) results for 100 Vpp and 200–700 Hz, with 50 Hz steps (pilot psychophysical experiments); and (**f**) results for 150 Vpp and 200–700 Hz, with 50 Hz steps (psychophysical experiments presented in Section 3.4).

Spectral analysis showed coherence with the nominal stimulation parameters. For each stimulation amplitude (50, 100, and 150 Vpp, see Figure 9a–c) the encapsulated transducer showed coherency in the stimulus presentation within the whole examined frequency range, with evident vibratory changes across the analysed peak-to-peak amplitudes and frequencies. The same is visible in Figure 9d–f showing the frequency values selected for the psychophysical testing (range 200 Hz–700 Hz, with 50 Hz steps). These experimental results allow to conclude that the vibrotactile stimuli delivered to the participants were coherent with the frequency values selected for the experimental testing and, thus, the transducers embedded in the polymeric matrix can deliver vibrotactile information in a reliable manner.

### 3.4. Psychophysics Results

Results from psychophysical testing for all the experimental configurations are shown in Figure 4 and are reported hereafter.

**Figure 10:**
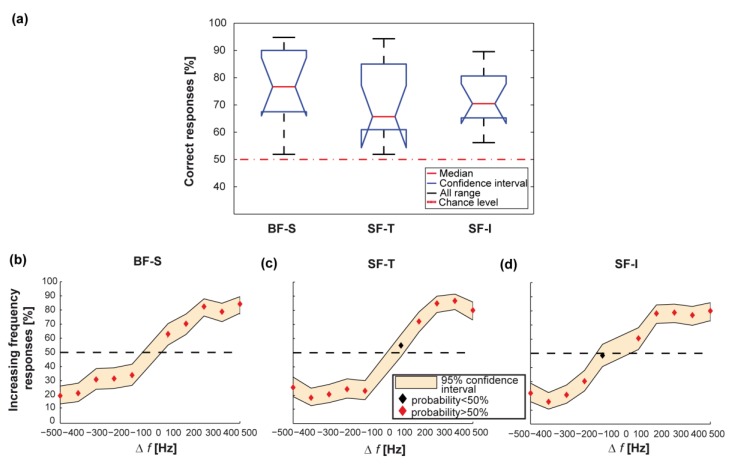
Stimuli perception with single-finger configuration. (**a**) Comparison between the fraction of correct responses (i.e., increasing frequency variations identified as increasing or decreasing frequency variations identified as decreasing) in all three configurations. Boxes represent the interquartile range and black dashed lines show the complete range across participants; (**b**) psychometric curve for the BF-S stimulation configuration. Each dot represents the fraction of times each stimulus was classified as having an increasing frequency (median across participants). If the identification rate is significantly different (average > 50%, binofit test) from chance the dot is red, otherwise, it is black. The filled area indicates the 95% confidence interval (binofit test) across participants and the black horizontal dashed line represents chance; (**c**) the same as (b) for the SF-T stimulation configuration; and (**d**) the same as (b) for the SF-I stimulation configuration.

The average performance over all the 10 frequency variations was significantly above chance for both bi- and single-finger configurations, with non-significant differences between the mean discrimination performances achieved under different stimulation configurations (as shown in Figure 10a: *n* = 11 for every group; 77 ± 11% for BF-S vs. 70 ± 8% for SF-I vs. 66 ± 12% for SF-T; *p* = 0.51, Kruskal-Wallis test). In particular, frequency differences larger than 100 Hz were reliably identified in all configurations (Figure 10b–d).

The developed integrated haptic system was thus effective in delivering vibrotactile information for both single-finger and bi-finger configurations when the frequency delivered to the skin was within the 200–700 Hz range and the stimulus variation was larger than 100 Hz.

Furthermore, in the explored frequency variation range (Δ*f* = −500–500 Hz) the psychometric curves obtained from experimental data for single-finger configurations, as well as the one for bi-finger configuration, were accurately fitted by logistic curves over the whole range of frequency variations (Figure 10b–d), $\chi^{2}=0.43$ for BF-S, $\chi^{2}=0.68$ for SF-I and $\chi^{2}=1.02$ for SF-T).

When calculating the psychometric curves to evaluate performance as a function of frequency variation, the differences between bi-finger and single-finger configurations showed that two-digit perception has a frequency sensitivity to vibrotactile stimulation comparable to the one relative to single-finger perception (compare Figure 10b with Figure 10c,d).

**Figure 11:**
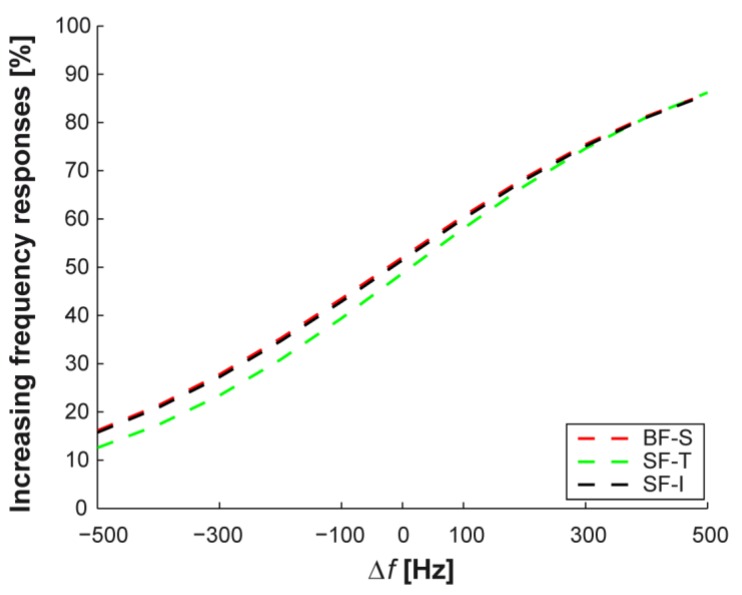
Comparison of the logistic fit of psychometric curves for all configurations. Comparison of logistic fit curves over the whole range of frequency variation. The curves are similar for all three experimented configurations.

For all configurations, Figure 11 shows the comparison between logistic fits of frequency variations identified as increasing. Over the full range of frequency variation, the two single-finger configurations and the bi-finger configuration had similar logistic fit curves. 

## 4. Conclusions

### 4.1. Main Results of the Study

In this work we (1) described the mechanical behaviour of a piezoelectric disk encapsulated in a polymeric matrix, specifically designed for the integration in wearable haptic displays, and (2) characterized the behavioural performance in sensory discrimination of healthy human subjects wearing such displays.

The FEM modelling showed that the normal force exerted by the encapsulated element presents a constrained variation across the experimented frequency range for the three selected driving voltages. This modelled behaviour was confirmed by the experimental testing.

The psychophysical testing of an integrated haptic system, a vibrotactile glove for the stimulation of the index and/or thumb fingertips of the human hand, demonstrated that the developed haptic transducer is effective in delivering vibrotactile information.

The transducer described herein presents some advantages with respect to the recent technological solutions for wearable tactile displays. The piezoelectric actuator enables the selection of a wide range of stimulation frequencies in the perceptual frequency range with a fine selection of frequency steps. This is true especially when piezoelectric elements are compared to polymeric actuators, like dielectric elastomers. The former allows selecting frequencies in both low and high frequency ranges. They can be used for the stimulation of both slowly-adapting and fast-adapting tactile receptors, in particular Pacinian corpuscles more sensitive to vibrotactile stimuli. Piezoelectric disks also limit the space required by the actuator, enabling a straightforward integration in wearable systems like gloves, and provide appropriate spatial resolution for being placed on different areas of the skin. These advantages are evident when our solution is compared to tactile actuators like pneumatics, vibrating motors, solenoid,s and to exoskeletons. In fact, a wearable device should fit the wearer’s body and allow the natural movements of the body part on which it is applied. When worn on the hands, tactile actuators must be lightweight and have limited dimensions [27]. The introduction of a polymeric shell to encapsulate the active element allows obtaining an integrated actuator which can easily fit in a wearable system for the stimulation of different body areas. The polymeric encapsulation also behaves as an interfacing layer between the piezoelectric element and the user’s skin, and our particular geometry allows the selection of a precise contact site to stimulate the insulation of the electrical connections increasing the actuator safety, and obtain an easy-fitting element.

### 4.2. Potential Applications of Multi-Finger Vibrotactile Stimulation

The transducer presented herein, thanks to its particularly shaped polymeric encapsulation and its broad range of actuation frequencies, can be effectively employed for the development of tactile displays suitable to different application scenarios. This encapsulated piezoelectric transducer can be integrated within tactile displays for sensory substitution to assist the blind or visually-impaired, deaf or hearing-impaired, and combined sensory impaired (deaf-blind) individuals. Several examples can be found in the literature about tactile aids used to convey the information coming from one sensory channel to the tactile sense in a perceptible manner [3,4,5,7,49].

Other applications can involve the integration of our encapsulated actuator in devices providing non-invasive tactile feedback to amputees. It can be also integrated in sensory augmentation technologies for healthy subjects in applications such as virtual reality, gaming, rehabilitation, navigation, rescuing, and remote control of robots, where vibrotactile stimulation is already widely used [22,23,24,25,26]. Haptic feedback also has significant advantages in robotic surgery and industrial environments for human-robot co-working activities. We are also considering the possibility to integrate this encapsulated actuator in haptic wearable systems for alerting purposes, in environments where the interaction with automated machinery can be dangerous for operators. This solution may improve safety in dangerous workplaces and the adoption of collaborative robotics in common applications.

Towards deployment in such scenarios, this study will be complemented with future experiments where these novel vibrotactile transducers can be integrated into different wearables, according to the particular application they will be involved in.

## Figures and Tables

**Table 1:** Experimental stimulation parameters. Ten pairs of vibrotactile stimuli. First vibrotactile stimulus: *f_1_*; second vibrotactile stimulus: *f_2_*; and related frequency variation ∆*f* = *f_2_* − *f_1_*.

Vibrotactile Stimulus	Vibrotactile Stimuli									
***f_1_* (Hz)**	700	650	600	550	500	400	350	300	250	200
***f_2_* (Hz)**	200	250	300	350	400	500	550	600	650	700
**∆*f* (Hz)**	−500	−400	−300	−200	−100	100	200	300	400	500
